# Using scenario videos with Theatre Testing method to adapt a peer navigation model to improve street-connected youth's access to HIV care in Kenya and Canada

**DOI:** 10.3389/fpubh.2022.975117

**Published:** 2022-11-03

**Authors:** Katie MacEntee, Edward Ou Jin Lee, Abe Oudshoorn, Alex Abramovich, Reuben Kiptui, David Ayuku, Amy Van Berkum, Olli Saarela, Thai-Son Tang, Edith Apondi, Juddy Wachira, Sue-Ann MacDonald, Paula Braitstein

**Affiliations:** ^1^Dalla Lana School of Public Health, University of Toronto, Toronto, ON, Canada; ^2^Centre for Addiction and Mental Health, Institute for Mental Health Policy Research, Toronto, ON, Canada; ^3^École de Travail Social, Université de Montréal, Montréal, QC, Canada; ^4^Arthur Labatt Family School of Nursing, Western University, London, ON, Canada; ^5^Department of Psychiatry, University of Toronto, Toronto, ON, Canada; ^6^Academic Model Providing Access to Healthcare in Eldoret, Eldoret, Kenya; ^7^Department of Mental Health and Behavioral Sciences, School of Medicine, Moi University, Eldoret, Kenya; ^8^Moi Teaching and Referral Hospital, Eldoret, Kenya; ^9^Department of Epidemiology and Biostatistics, School of Public Health, Moi University, Eldoret, Kenya

**Keywords:** Theatre Testing method, HIV Peer Navigation, street-connected youth, Kenya, Canada

## Abstract

Theatre testing (TT) method demonstrates whole or portions of an evidence-based intervention to stakeholders to elicit feedback on context-specific adaptations and future implementation. The Peer Navigator Project (PNP) studied the adaptation and implementation of Peer Navigators in five urban sites to increase street-connected youth (SCY) access to HIV prevention, testing, and treatment in Canada and Kenya. TT was used with SCY, healthcare providers, and community stakeholders to collect feedback on the optimal characteristics of the PNs (e.g., social identities) and their professional activities and responsibilities in each site. Sites scripted unique scenarios of PNs supporting SCY and interacting with social service providers. Local actors were employed, and the scenarios were filmed and edited into videos alongside audience discussion questions. Videos were screened to separate audiences of SCY (*n* = 40), healthcare providers (*n* = 12), and community stakeholders (*n* = 59). Facilitated discussion about the scenarios were recorded as data, and transcripts were analyzed thematically by the research team. The scenario videos are presented as a unique adaptation to the TT method. The adaptations were time-consuming and limited the ability to present responsive changes while presenting the method to different audiences. They were also effective at maintaining presentation fidelity and eliciting diverse and meaningful responses from different stakeholder groups. One site successfully adapted the method for use in a physically distanced manner that complied with COVID-19 public health regulations. TT using video scenarios is an engaging approach that garners rich responses from diverse stakeholder groups about the adaptation of evidence-based interventions preparing for implementation in international settings.

## Introduction

The Theatre Testing (TT) method has been proposed as a strategy that can be used outside of a randomized control trial and with clinical and community-based study populations to adapt evidence-based interventions and gauge reactions of the target population to new interventions (1). Live-action demonstrations of core elements of an intervention are presented to key stakeholder groups (e.g., youth and service providers). Facilitated group discussions record feedback on intervention components. The method's strength is in its ability to mimic intervention implementation to stimulate highly accurate participant responses, especially related to how intervention content, materials, and delivery style should be adjusted to increase relevancy and efficacy for use with a target population (1).

TT has been implemented with various populations to explore HIV and other health related topics. It was used to assess a mobile app to support uptake of HIV testing, counseling and pre-exposure prophylaxis amongst young men who have sex with men (2, 3) as well as to prepare an HIV intervention for implementation with African American and LatinX young women (4). More broadly, TT has been used by studies on: sensitizing caregivers and teens on matters relating to LGBTQ+ youth (5); supporting Hmong-American parents (6); managing general anxiety disorder amongst students in India (7); telemonitoring equipment for Type 2 Diabetes (8). A review of practice guidelines for adapting evidence-based population-based health initiatives found TT to be an effective technique for engaging different populations in responding to proposed intervention adaptations (9).

To the best of our knowledge, creating scenario videos depicting intervention implementation has not been used with TT method. One study screened and recorded feedback on videos as educational tools to learn how to use intervention equipment (8). In this paper, we present how the TT method was adapted and implemented. We describe how video scenarios depicting the implementation of a Peer Navigator (PN) intervention were created. We further describe how the videos were used to garner feedback from diverse stakeholder groups to identify the site-specific adaptation needs in preparation to implement a PN that would support street-connected youth (SCY) to access HIV prevention, testing, and care. We explore how the video scenarios were developed and employed to collect data prior to and during the COVID-19 pandemic. The use of video scenarios is discussed as a unique adaptation and thus contribution to the TT method that engages diverse and marginalized participant groups in providing rich feedback on evidence-based intervention adaptations.

## Study setting: The Peer Navigator Project

PNs share similar characteristics (e.g., HIV status, age, race) and lived experiences (e.g., experience with homelessness) with their target population, to whom they provide navigational support with healthcare engagement. The Peer Navigator Project (PNP) used TT to study the adaptation of a Peer Navigation model (10, 11) to increase SCY (ages 16–29) access to HIV prevention, testing and treatment in five sites—Montreal, Toronto, and London, in Canada, and Eldoret/Huruma and Kitale, in Kenya. In Montreal and Toronto, the study prioritizes SCY who identify as Two Spirit, Lesbian, Gay, Bisexual, Trans, and Queer (2SLGBTQ+). In the other three sites, youth of any gender identity or sexual orientation participated. A partner organization in each site is responsible for hiring one PN. In preparation for hiring, TT was used to show components of the intervention in action in a simulated context and invite participant feedback with the goal of clarifying optimal PN characteristics (e.g., HIV status, race, age, gender identity, sexual orientation) and their core roles and responsibilities in each partner organization. This project received ethical approval from the University of Toronto, Moi University, Centre for Addiction and Mental Health, and Université de Montréal.

### Participants

Three groups of participants were recruited for involvement with the TT activities: SCY, healthcare providers, and community stakeholders (e.g., case workers, housing support workers). It was important that those participating in the TT were consistent with those who would also be involved with the actual intervention implementation. Study inclusion required healthcare providers and community stakeholders to have at least 3 years of experience working directly with SCY. Youth participants were required to self-identify as street involved and/or homeless and be between 16 and 29 years old. The inclusion criteria in Montreal and Toronto also required youth identify as 2SLGBTQ+. Participation was anonymous and voluntary. All participants signed institutionally approved consent forms and received honorarium (CND30/KES1000).

## PNP Theatre Testing method

### Community consultation

In preparation for the TT, site teams conducted key informant interviews and focus groups to assess the acceptability of the PN intervention in each of the project sites. This process gauged stakeholder perspectives on current barriers and facilitators to SCY access to HIV and related health services (e.g., mental health, harm reduction). Participants also described the acceptability and perceived challenges of implementing the PN intervention in their site. A components checklist collected quantitative data from participants on the core and peripheral components of PN characteristics and responsibilities, as well as that of a PN employer. PN characteristics included personal, intersecting identifiers, including age, HIV status, and life experiences. PN responsibilities were activities that the PN might complete as part of their job, such as accompanying clients to appointments and accompanying clients into the clinical setting. The employer's responsibilities explored how best to support the PNs (e.g., supervision and support). Interview and focus groups transcripts were analyzed thematically (12). The preliminary results of this consultation informed the development of video scenarios that presented simulated PN interactions with SCY, healthcare providers and community stakeholders.

### Developing scenario scripts

The preliminary findings of the community consultation data identified areas of contestation or uncertainty regarding PN roles and activities (e.g., age, race, and gender, and how PNs interact with SCY and healthcare providers). PNP site teams crafted scenarios to invite specific feedback from the TT participants to address these contested aspects. For example, scenarios in the Canadian sites depicted PNs of different ethno-racial and gender identities and follow-up discussion questions prompted audience reflections on these characteristics. Toronto scripts were completed first and shared with the other sites to use as a template. Each site revised their scripts several times until the scenarios were deemed to be realistic, depicted a range of SCY engagement strategies, and depicted the PNs interacting with service providers. This included representing intersectional stigma toward PN and SCY from healthcare providers based on homelessness, mental health, HIV, and gender identity as well as the complexities of providing peer support to serodiscordant couples and individuals who are unhoused and pregnant (Table 1). In Montreal the partner organization contributed to the concept development and scenario approval process. Four scenarios were created for use across all the Kenyan sites. The Canadian sites produced three scenarios each (*n* = 6). Scripts were written in the dominant local languages (Kenya = Swahili; Toronto and London = English; Montreal = French, Spanish, and English).

**Table 1:** Summary of TT scenarios.

**Site**	**Scenario 1**	**Scenario 2**	**Scenario 3**	**Scenario 4**
Toronto	The PN arranges emergency shelter and a doctor's appointment for a trans youth who is living with anxiety and HIV. When the youth does not follow the PN referral the PN accompanies the youth to their medical appointment. The PN advocates on behalf of the youth to receive further health services from the doctor.	A PN accompanies a SCY for an HIV test. The healthcare staff exhibit cis-normative behavior. The PN educates the staff on gender non-binary protocol. The youth has an HIV test and the results are positive. The PN supports the youth in coming to terms with living with HIV.	A new PN approaches a couple at a drop-in center. The couple is serodiscordant. The PN encourages the couple to go for HIV testing and, after some discussion, they agree.	N/A
London	A PN supports a non-binary youth who is at risk of contracting HIV, but afraid to get tested. The youth decides to get tested and discovers they are HIV+. The PN supports them to process this new diagnosis.	A PN encounters a youth they have a working relationship with at a popular youth café. The PN and the youth, alongside the youth's partner who is living with HIV, discuss HIV testing and prevention.	A PN conducts street outreach and meets with a female youth who is HIV+, struggling to find secure housing, and experiencing challenges with mental health and substance use. The PN supports the youth to re-engage with HIV treatment and find other support.	N/A
Montreal	The PN meets with a trans youth living with HIV in the street and shares information related to emergency shelter resources and coordinates a doctor's appointment. The PN also accompanies the youth to their medical appointment. The PN advocates on behalf of the youth to receive further services (especially mental health services) from the doctor. Language: English	A PN approaches a couple (trans woman and cis man) at a SIY drop-in center. The cis man is HIV positive, the trans women last did an HIV test 2 years ago and tested negative. The PN provides information about PrEP and encourages the couple to go to accessible HIV testing and, after some discussion, they agree. Language: French	A PN accompanies a SIY for an HIV test. The SIY uses she/her pronouns, and the healthcare staff exhibit cis-normative behavior (i.e., misgendering). The PN educates the staff on gender non-binary protocol. The youth's HIV test result is positive. The PN supports the youth in coming to terms with living with HIV. Language: Spanish, with some English [NOT FILMED DUE TO COVID-19 HEALTH RESTRICTIONS ON IN-PERSON GATHERING]	N/A
ELDORET, Huruma and Kitale	A heterosexual couple talks to a PN and agree to go for HIV tests.	An SCY is symptomatic but reluctant to take HIV treatment. The PN explains the benefits of treatment.	A SCY is newly diagnosed and experiences challenges staying on treatment. The PN refers to support services.	A PN talks to a pregnant SCY about the benefits of staying on ARV treatment while pregnant and breastfeeding.

### Filming

The research team intentionally arranged filming of the scenarios to avoid appropriating or misrepresenting SCY and their experiences. Sites hired a mix of professional and untrained local actors and community filmmakers with lived experience with housing insecurity and/or HIV. In Toronto and Montreal, this also included actors and filmmakers who identified as 2SLGBTQ+. Actors were also from diverse ethno-racial backgrounds. In most of the sites, actors with lived experiences improvised off the scripts based on their knowledge of the topic. In Montreal, the director worked closely with the cast in preparation for filming and improvisation was not encouraged. Research team members were present during the filming to provide directorial guidance. Actors and filmmakers were paid, and the videos were shared so they could be included in professional portfolios. Engaging local community strengthened the authenticity of the scenarios and contributed to employment opportunities for young people, many of whom may struggle with low and under employment given intersecting social stigma and marginalization. Each site filmed 2–4 scenarios (*n* = 12). Montreal filmed two scenarios, but public health measures early in the COVID-19 pandemic did not allow the filming of their third scenario. After editing, scenarios ranged in duration between 3 and 7 min.

### Theater Testing videos

The scenarios were combined into TT videos (*n* = 4). London and Toronto created unique videos, each containing three scenarios. Montreal used 2 scenarios, one in French and one in English. Kitale/Huruma and Eldoret sites used the same video with four scenarios. Toronto, London, and the Kenyan sites embedded their discussion questions into the video following the scenarios. Subtitles and voice over were added to the videos to increase their accessibility. In Montreal, participants were provided with English/French scripts as required in lieu of subtitles.

### Screenings

Participants attended a single screening of the TT videos. Audiences were groups of 5–17, with separate screenings being organized for SCY, healthcare providers, and community service providers; although, there were service providers who also identified with lived experience of being street-connected. Separation into distinct viewing groups was to address potential power inequalities between youth and adults that might hinder participation and full, honest feedback.

Screenings lasted 1–1.5 h. In most sites, participants and one or two members of the research team met in-person. In Montreal, screenings were conducted over Zoom due to COVID-19 restrictions. Engagement was in English in Toronto and London, Swahili in the Kenyan sites, and French, English, or a mix in Montreal. After a brief introduction to the PN project and review of the theatre testing method, participants completed the REB-approved consent process. In Montreal, English and French transcripts were distributed as language aids for mixed-language groups and participants were also provided with a description of the PN role. Across sites, scenarios were screened one at a time. After each scenario, a 10–15 min facilitated discussion of participants' responses to the video discussion questions and general reactions about each scenario were audio-recorded and later transcribed.

### Data analysis

Transcripts were coded and then thematically organized using NVivo qualitative analysis software. To construct the codebook, seven researchers from the Canadian and Kenyan sites read one transcript from the three stakeholder groups to identify dominant nodes. Separate codebooks were created in both countries and then compiled. Each code was defined and approved by the team. The team piloted the compiled codebooks using two transcripts (one Canadian and one Kenyan) and minor revisions to code hierarchy and definitions were applied. A team of coders (graduate research associates, a postdoctoral fellow, and project manager) then read and coded all the transcripts. The team met regularly to maintain consistency in the coding process. Subsequent rounds of coding resulted in minor changes to the codebook and the generation of theme memos. Coded data went through two rounds of review by the coding team to ensure consistency across sites. Themes were identified in relation to PN characteristics, defining the PNs' roles within their host organizations, and identified challenges associated with intervention implementation. Findings informed the development of the PN job descriptions, trainings, and their integration into the project's partner organizations—all key aspects of the subsequent intervention implementation.

## Findings

### In-person and virtual participation

A total of 112 participants took part in the TT method. A breakdown of the participants by participant group by site is presented in Table 2. No further demographic data were collected. The COVID-19 pandemic greatly impeded participant recruitment. Prior to the pandemic, London completed all its TT activities and screenings were conducted in-person with healthcare providers, community stakeholders, and SCY. In Toronto, screenings were completed in-person with community stakeholders and SCY. In Kenya, screenings were completed in-person with healthcare providers and SCY. Eldoret, Huruma, and Toronto were unable to recruit healthcare providers after the onset of COVID-19. Healthcare providers and community stakeholders experienced unprecedented demands on their time and participation in TT activities became unfeasible. The Montreal site completed TT data collection during COVID-19. They obtained an ethics amendment to hold TT screening activities online and hosted 6 virtual screenings using Zoom with community stakeholders and healthcare providers. No SCY participated in this site, although some of the community stakeholders had previous lived experience as LGBTQI+ SCY and reflected on the video scenarios from this perspective. The video scenarios (filmed prior to the pandemic) made transitioning to the online platform easy, especially in the urban Canadian setting where internet access is reliable and widespread. There was no perceived shift in the quality of the participant engagement online compared to in-person. The virtual format facilitated different levels of participant-controlled anonymity in the focus group setting. They controlled whether they wanted to show their face or participate using audio-only and/or using the chat function. Zoom's built-in record function allowed facilitators to record the verbal discussions and the chat was also saved for analysis.

**Table 2:** Theatre Testing participants.

**Participant group**	**Toronto**	**Montreal**	**London**	**Kitale**	**Eldoret/Haruma**	**Total**
Youth	6	0	10	12	12	40
Healthcare	0	5	7	0	0	12
Community	10	16	9	12	12	59
**Total**	**16**	**21**	**26**	**24**	**24**	**111**

### Interpreting participant video responses

Participants had different reactions to the video and were eager to contribute to discussions. They made direct reflections on the videoed scenarios. For example, one participant shared, “I liked the part where they said, ‘I'm worried about you' it shows that the person is there for them,” (Montreal, healthcare provider). These types of direct reflections provided clarity on a broad range of PNs characteristics—in this case, empathy. Another participant stated, “she was very calm and did proper counseling to the client. She even told the client she's also positive but living positively this made the client to calm down,” (Eldoret, community stakeholder). This type of feedback also described optimal PN characteristics (being calm and HIV+) alongside indication of critical PN trainings (counseling for those newly diagnosed). Participants also compared scenarios to illustrate the strengths and weaknesses of different PN approaches, “I think what helped in this scenario is that the PN talked about his background, his experience and about himself, which the PN in the first scenario did not do. I think it helped build trust,” (Montreal, healthcare provider). The emphasis on PNs' willingness to share personal information to support relationship building with youth is interpreted alongside other audience responses that stressed the importance of PNs maintaining professional boundaries.

Participant A: “I saw that she used touch which can be really effective but it's not something that they will want upon them. So, I think that can be a little dicey.”

Participant B: “I found that troubling too like given that case reviewers are supposed to teach like peer workers how to work with people. Boundaries are an important part of that. So, I don't think that touch is a wise thing to use in these videos,” (London, Community Stakeholder)

A PN must balance sharing their peer status alongside their professional responsibilities as a care provider. The videos also elicited personal disclosures amongst the participants. In response to a scenario depicting a doctor's treatment of an SCY, one participant shared:

I've also gone through something similar with a doctor due to my drug use… I lived through all of that, and when I got out of there, I was more angry than anything. So, I really liked how the worker wanted to accompany the youth throughout this process and into the doctor's office, I think it's a good thing, (Montreal, community stakeholder)

Personal reflections such as this validated that the scenario content was relevant to the PN contexts and further validated participants' feedback as coming from a knowledgeable, experienced source. Some community stakeholder participants spoke from their perspective as current service providers and past service users. Responses offering these dual perspectives were especially relevant in sites that had fewer or no SCY participants.

Participant responses also indicated problems with the scenarios that glossed over the challenges of PN work. As one participant put it, “you really need to find those personalities that are willing to sort of get down and dirty because that [video scenario] was cute but it's never going to go that way,” (Toronto, SCY). This participant's feedback, while suggesting that the scenario was unrealistic, helpfully gave attention to the importance of the PN being committed to the job and activities that accommodate meeting SCY where they are at. Thus, even negative feedback on the scenarios provided valuable feedback about PN characteristics and roles. Overall, the scenario-based TT approach provided rich, multifaceted reflections on PN characteristics as well as information that elucidated the PN role and responsibilities.

### PN characteristics

Participants made direct reflections on PN characteristics. For example, one participant suggested, “I think commonality is important, like for example knowing that the other person's also queer, for me like if I had a peer navigator who was also autistic or also had anxiety or both I would instantly want to listen to them more because I'd feel like they understood,” (Toronto, SCY). Another participant pleaded, “Please people of color, like there's a severe lack of people of color,” (Toronto, SCY). These types of comments helped the research team to better understand how to strategically choose PN characteristics to increase the diversity amongst service provider staff in some sites.

There was no consensus on the PN characteristics. Several participants noted a project limitation to be its funding to hire just one PN in each site. Ideally, there would be multiple PNs who could connect with the diversity of SCY and as one participant explained, “somebody to choose who they want to get support from,” (London SCY). Nevertheless, the discussions identified how PN characteristics might be leveraged to target SCY who are especially marginalized from services and supports. In Kenya, it was the lived experience of homelessness and knowledge of the SCY communities that was viewed as the most pertinent PN characteristics. As one participant described, “these guys are very hard to find, going out there to look for them helps so much in delivery of service,” (Eldoret, community stakeholder). The age of the PN was a topic of discussion across sites. Some participants worried that a younger PN might struggle to connect with older youth and with service providers. SCY participants in London felt that an older PN would be more respectful, trustworthy, and would be less likely to break their confidentiality. In London and Toronto, participants suggested that the PN should identify as trans, as this would promote a peer connection between an underserved population of SCY who are especially at risk of experiencing violence. However, London healthcare providers suggested that a man or woman PN would likely engage clients of the same gender and young women would be less comfortable connecting with a PN who identified as a man. This was especially relevant should the PN seek to connect with girls and young women who are being trafficked for sex.

### PN skills

PN skills were sometimes considered more important than characteristics:

“I don't know that gender particularly matters, it's the individual's capacity to anticipate and respond accordingly to situations that might arise,” (London, healthcare provider).

Likewise, another participant stated, “I don't think [race is] a qualifier either way, because I think your response to a situation is more important than what you look like” (London, healthcare provider). In Eldoret, participants reported that PNs should speak in an accessible language to facilitate comprehension of complex health issues. Other skills that participants described across sites included: being able to exercise good judgement and de-escalate tense situations, being a good listener, having good people skills (e.g., being easy to talk to and being able to sit comfortably in silence), and, as previously mentioned, being empathetic and able to maintain clear boundaries. Having knowledge of the service network in the site was a highly valuable skill, especially if it allowed the PN to refer SCY to supports that were accessible, non-judgmental, and reliable. Participants also described the importance of PNs being reliable, which one participant defined as “showing that you're just not a one-off, like you constantly engage in the conversation, like maybe not specifically day after day but over a period of time,” (Toronto, SCY).

Overall, the screenings prompted a wide array of responses from participants and evoked rich reflections on the implementation of PNs in each site. This led the PNP team to explore the broader pedagogical value of the TT videos by incorporating them into the subsequent PN trainings. Having watched the videos independently, PNs discussed scenarios as a group, identified problems with the PNs activities in the scenario, and reflected on how they might respond differently. The videos were later incorporated into subsequent PN trainings. This involved PNs watching the scenarios independently, then coming together to discuss the scenarios as a group, including identifying what were the strengths/weaknesses of the PN interactions depicted in the scenarios, and reflecting on how they might respond differently in a similar situation. In the Montreal site, partner organization staff were also involved in the video-based training.

## Discussion

Key stakeholder involvement is critical to informing intervention adaptation and strengthening the likelihood of intervention success in a particular site (9). Adapting the TT method to use video scenarios required considerable commitment from the PNP team, and in Montreal the partner organization, to organize. However, the results of these efforts were rich. This echoes previous research that has used TT to collect feedback from diverse participants on the adaptation of evidence-based HIV interventions targeting systemically marginalized populations (1, 3, 4). In this section, we discuss how the PNP's use of the video scenarios contributes to the development of TT method as it relates to: (1) The scenario-based approach, (2) Participant engagement, and (3) The video-based approach.

### The scenario-based approach

A strength of the TT approach is its ability to demonstrate interventions (in their entirety or specific segments) to garner robust feedback from key stakeholders about adaptations (4, 7). The complexity of SCY lives and the myriad ways that PNs might respond and support SCY navigate their healthcare access posed a considerable challenge for the PNP when figuring out exactly how to depict the PN role to TT audiences. Community consultations and partner organization involvement in scenario development were critical for recognizing this diversity in potential implementation and identifying specific areas of adaptation concern for each site. Presenting a standardized PN interaction with a SCY (should one even exist) was deemed impossible. Rather, we created scenarios that were believable, but could also be viewed as flawed. Depicting different interpersonal engagement strategies prompted audiences to reflect upon PN characteristics and skills. Ensuring discussions were open-ended allowed participants to shed light on unanticipated topics. The generative discussions, especially those tied to personal reflections and those that dissected the micro-processes of relationship-building and service provision, was associated with Pablo Freire's pedagogy of problem-based learning (13). This included the conscious involvement of participants by inviting them to apply their lived experiences to identify problems in the scenarios that connected to larger challenges in providing support services to SCY and discussed alternative, more appropriate methods of support.

### Participant engagement

Audience feedback is potentially hindered by inter- and intra-group power dynamics. To encourage feedback from their mixed stakeholder audience, Wingood and DiClemente encouraged active engagement with the demonstrations from their targeted youth audience members and more passive observation followed by feedback from the adult stakeholders in the audience (1). The PNP strategy was to separate audiences into key informant groups. Keeping audience groups separate meant discussion between groups was not possible. Nor did it avoid potential intra-group exclusion within stakeholder groups. Still, the rich responses to the screenings suggest that individuals felt comfortable sharing their ideas. Facilitators with experience leading focus group discussions and working with the target populations were invaluable, especially when seeking responses from quitter participants and when intra-group dynamics was a concern (e.g., one participant is dominating the discussion). Supporting more equitable participant engagement had the unanticipated and welcome outcome of introducing and raising awareness of the intervention and the soon-to-be hired PNs in the sites.

### The video-based approach

TT methods that use live-action performances of interventions can be performed several times to the same or different audiences (1, 3, 4). This allows actors to “pilot” audience feedback immediately, in front of participants, to check understanding and to explore emerging ideas. However, how might unplanned performance variations impact audience feedback? The PNP adaptation of using pre-recorded scenario videos meant all audiences in the same site saw the same depiction of PN activities, ensuring fidelity between screenings. Furthermore, video production stimulated employment opportunities for the disenfranchised communities of youth our project seeks to support. Being able to show the videos repeatedly was cost effective and there was value added when they were later used during PN training. Videos made shifting from in-person and online screenings relatively straightforward. In contexts where the internet was reliable and accessible, virtual screenings supported the participation of busy stakeholders. Using Zoom offered greater means of anonymous participation (e.g., keeping their camera off) and increased accessibility (e.g., use of chat, closed captioning). The downside was that adaptations or updates to the video scenarios to evolve with ongoing feedback would be time consuming and costly. Virtual screenings may be less feasible in contexts where internet access is inconsistent and/or unpredictable. SCY may have limited access to Internet or lack a private space in which to participate in discussions on sensitive or personal topics. Future projects might consider how to mitigate these limitations by sourcing private viewing areas, covering costs of internet access, and supplying needed technology to participants.

## Conclusions

We have presented an adaption to the TT method that included crafting unique scenarios and using video to elicit stakeholder feedback on a PN model requiring adaptions to support SCY access HIV care. The video scenarios were highly engaging and produced rich data that illuminated site-specific adaptions to PN characteristics and skills. The video-based TT approach was applicable in diverse locations. It supported in-person and online engagement of diverse participant groups. When deciding between using in-person or pre-recorded demonstrations of an intervention, future projects should carefully consider participant access barriers and recognize that online sessions, when internet and private space is available, offers participants multiple ways to engage in discussion. Holding screenings for segregated stakeholder groups can help mitigate potential power inequities between participant members. We recommend having seasoned facilitators run the screening workshops so that participants are comfortable engaging in the semi-structured, open-ended discussions.

## Data availability statement

The raw data supporting the conclusions of this article will be made available by the authors, without undue reservation.

## Ethics statement

The studies involving human participants were reviewed and approved by University of Toronto, Moi University, CAMH, and Université de Montréal. The patients/participants provided their written informed consent to participate in this study.

## Author contributions

KM was centrally involved in the adaptation of the TT method and led this paper, from conceptualization through drafting and revisions. PB is the principal investigator on the project. All other authors listed contributed to the data collection and provided feedback and suggested revisions of earlier drafts of the manuscript. All authors contributed to the article and approved the submitted version.

## Funding

This research was supported by an HIV Implementation Science Component II grant from the Canadian Institutes of Health Research, Institute of Public and Population Health.

## Conflict of interest

The authors declare that the research was conducted in the absence of any commercial or financial relationships that could be construed as a potential conflict of interest.

## Publisher's note

All claims expressed in this article are solely those of the authors and do not necessarily represent those of their affiliated organizations, or those of the publisher, the editors and the reviewers. Any product that may be evaluated in this article, or claim that may be made by its manufacturer, is not guaranteed or endorsed by the publisher.
